# TTSurv: Exploring the Multi-Gene Prognosis in Thousands of Tumors

**DOI:** 10.3389/fonc.2021.691310

**Published:** 2021-05-25

**Authors:** Yue Qi, Mengyu Xin, Yuanfu Zhang, Yangyang Hao, Qian Liu, Peng Wang, Qiuyan Guo

**Affiliations:** ^1^ College of Bioinformatics Science and Technology, Harbin Medical University, Harbin, China; ^2^ Department of Gynecology, The First Affiliated Hospital of Harbin Medical University, Harbin, China

**Keywords:** noncoding RNAs, thoracic malignancy, prognostic biomarkers, high-throughput analysis, biological database

## Abstract

Thoracic malignancies are a common type of cancer and area major global health problem. These complex diseases, including lung cancer, esophageal cancer, and breast cancer, etc. have attracted considerable attention from researchers. Potential gene-cancer associations can be explored by demonstrating the association between clinical data and gene expression data. Emerging evidence suggests that the transcriptome plays a particularly critical role as a diagnostic biomarker in pathology and histology studies. Thus, there is an urgent need to develop a platform that allows users to perform a comprehensive prognostic analysis of thoracic cancers. Here, we developed TTSurv, which aims to correlate coding and noncoding genes with cancers by combining high-throughput data with clinical prognosis. TTSurv focuses on the application of high-throughput data to detect ncRNAs, such as lncRNAs and microRNAs, as novel diagnostic and prognostic biomarkers. For a more comprehensive analysis, a large amount of public expression profile data with clinical follow-up information have been integrated into TTSurv. TTSurv also provides flexible methods such as a minimum p-value algorithm and unsupervised clustering methods that can classify thoracic cancer samples into different risk groups. TTSurv will expand our understanding of ncRNAs in thoracic malignancies and provide new insights into their application as potential prognostic/diagnostic biomarkers.

## Introduction

Thoracic malignancies are the most common type of cancers (1, 2), and their biomarkers have been the focus of medical research. In clinical studies, numerous genes have been strongly associated with the onset and progression of cancer lesions, usually manifested by the dysregulation of genes in the functional pathways of cancer. As research progresses, noncoding RNAs are gradually transforming from nonsense transcriptional products to regulators that mediate cellular processes, including chromatin remodeling, transcription, post-transcriptional modifications, and signal transduction. The versatility of noncoding RNAs allows them to participate in multiple biological processes and influence many different molecular targets. Therefore, noncoding RNAs are considered key regulators of physiological programs during the development of disease. Mutations and the dysregulation of noncoding RNAs play a critical role in cancer (3). Based on clinical information, prognosis, as a measure of genes in cancer, is often recognized as the most critical factor (4). By combining clinical patient survival data with their gene expression data, we are able to target genes whose expression is highly correlated with survival, and many experiments have demonstrated the potential of such genes as biomarkers for cancer diagnosis in general. For example, hsa-mir-196b and Nek2 are specifically expressed in patients with pancreatic ductal adenocarcinoma (5); RP13-30A9.2, RP11-488I20.9, and other genes are specifically expressed in patients with esophageal squamous cell carcinoma and has prognostic value (6); and decreased expression of ALDH5A1 predicts prognosis in patients with ovarian cancer (7). Currently, gene expression data and survival data for related samples have been published in several public databases and this information can be downloaded by researchers. The integration and analysis of these two types of data will yield more valuable information and broaden the scope of cancer research. In addition, a more comprehensive and objective conclusion can be obtained by comparing the survival analysis results obtained for the same gene in different datasets. However, there is an urgent need for an efficient data processing platform that can effectively analyze and process the increasingly large amounts of data.

When undertaking a survival analysis, it is often necessary to group patients according to certain metrics and compare the risk differences between groups, but the different cutoff values have important implications for the outcome, and there is currently a lack of a gold standard for cutoff values. Several databases exist that can be used to conduct a survival analysis of the expression profiling data, such as Kaplan-Meier Plotter, LOGpc, and OncoLnc online tools, but there is still a need for a biologically meaningful cutoff value. Users often differentiate samples using thresholds, such as median/trichotomies/quartiles, which are merely mathematical and do not express biological properties, or they calculate the division values multiple times, which is often time-consuming and lacks a scientific basis. Classifying samples multiple times and performing the Cox test can determine the optimal grouping and find the best separation point among continuous variables. It has been shown to be useful in the analysis of tumor size (8, 9), cell cycle phase estimation measurements (10), and gene copy number (11). Most data-dependent segmentation methods (e.g., mean, median, and quartiles) may not represent the true prognostic power of the predictors. However, the minimum p-value method uses statistical methods and clinical information guidance to systematically determine an optimal grouping value after grouping the sample multiple times. Therefore, we consider the cutoff value obtained by this method to be biologically meaningful (9) so we applied this approach to analyze expression profiles. In addition, the prognostic impact of genes on cancer patients can vary depending on the differences in the datasets selected by the researchers. For example, the choice of TCGA (12) data versus GEO (13) data can also have an impact on the analysis results, so a common test of multiple sample sets is usually required to produce results with higher confidence. In the current study and analysis, plotting survival curves alone does not provide strong evidence about the impact of changes in gene expression on patient survival. This suggests that more evidence is needed to illustrate the relationship between gene expression profiles and patient survival in multiple dimensions. Therefore, while providing different classification algorithms, we also provide a variety of visualization charts to better illustrate the results for users. For example, when multiple genes are passed into the same cancer dataset, we provide a forest plot and a correlation plot to visualize the relationship between all genes and when analyzing the survival of a single gene in cancer, we provide an integrated plot of the patient’s survival time, survival status, and the expression of that gene.

In summary, we have developed TTSurv. It is a large collection of cancer-related expression profiles and their associated clinical data that can be found in public databases such as GEO and TCGA. TTSurv also provides a minimum p-value method to calculate the best cutoff value for the user while allowing the user to manually submit the separation value. In addition, we classified the samples according to the expression value of the dataset and performed unsupervised tests. By providing multiple ways to classify samples, we can uncover all possible associations between genes and cancer. In addition, we also provide integration algorithms for multi-gene analysis to integrate the target gene set, which allows the user to analyze the entire gene set as a whole. Finally, the database provides an overall assessment of the prognostic value of genes in multiple datasets, which will gives researchers more experimental opportunities and more valuable analysis results.

## Materials and Methods

### Data Collection and Processing

**Figure 1:**
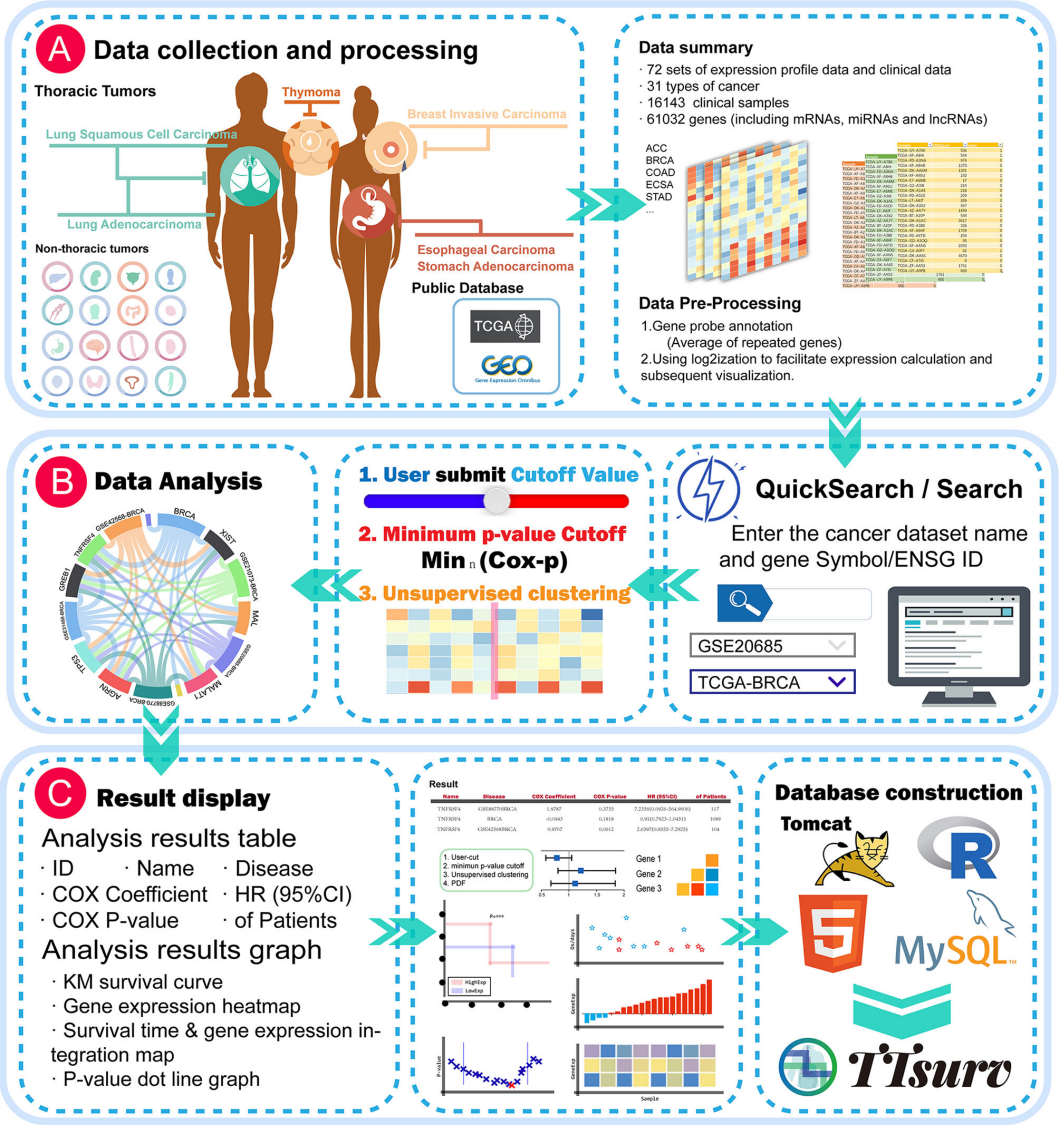
Global view of the TTSurv database. **(A)** illustrates the data sources and subsequent data processing by TTSurv; **(B, C)** show the user interface illustrating how the user searches for data and the results obtained; and **(C)** shows the results provided by TTSurv and its backend components.

Expression profiling data and survival data were obtained from the TCGA and GEO public datasets (**Figure 1A**). We applied the following selection criteria to further organize the data: 1. it contains sample prognostic information, 2.it uses a large sample size (sample size > 50), and 3. the probes of the platform are annotated with public identifiers (e.g., Gene Symbol, GenBank, UniGene ID, etc.). The expression profiles were derived from the series matrix files for each GEO dataset and log2-transformed. We collected 72 datasets containing 16143 samples from 31 cancers involving 61032 genes (**Tables S1** and **S2**). The probe-gene annotation information was derived from the GEO database, and each probe was mapped to an Ensembl-ID by querying the UniGene database for the accompanying public identifier.

### Minimum P-Value Algorithm

The minimum p-value method has been validated in clinical trials (14), and this method is also supported statistically (15). Accordingly, we used the minimum p-value algorithm to obtain the best grouping by undertaking multiple grouping and a survival analysis of both groups to filter the cutoff value corresponding to the minimum log-rank p-value.

First, patients were ranked according to the expression values of a given gene. Patients were then divided into two groups (high and low) at all potential cut-off points, and the difference in risk between the two groups was estimated using the log-rank test. The best cut point that gave the most significant p-value (P-minimum) was then selected (**Figure 1B**).

### Gene Integration Algorithm

The survival analysis, performed with multiple genes in a particular cancer, also provides a risk score model that considers the strength and the positive and negative associations between each RNA and survival probability. This means that we can assess the association between the expression of the entire set of genes and the survival of cancer patients. For each patient, the risk score was calculated by weighting the linear combination of all RNA expression values with the Cox regression coefficients.

$$Riskscore=\sum_{i=1}^{n}\beta_{i}\;Exp\;(C_{i})$$

where βi is the Cox regression coefficient for each RNA (denoted by C_i_), n is the number of RNAs in the gene set, and Exp(C_i_) is the expression value of RNA C*i* in the corresponding sample. Patients were classified into high expression and low expression groups according to the different classifications (16).

### Database Construction

The TTSurv online server was developed using Tomcat V7.0. We manually classified the data into miRNA expression profile data, and lncRNA and mRNA expression profile data. Each expression profile matrix was stored separately with its prognostic information in MySQL (V5.5). The data were visualized using Datatables, echart, highchart, and other plugins, and all statistical analyses were performed using the R framework (V3.6.0) (**Figure 1C**). TTSurv also supports the current mainstream browsers (e.g., Microsoft Edge, Google Chrome, Firefox, and Safari) and can be freely accessed at http://www.bio-server.cn/TTSurv.

## Results

### Diverse Pre-Result Presentation Interface

**Figure 2:**
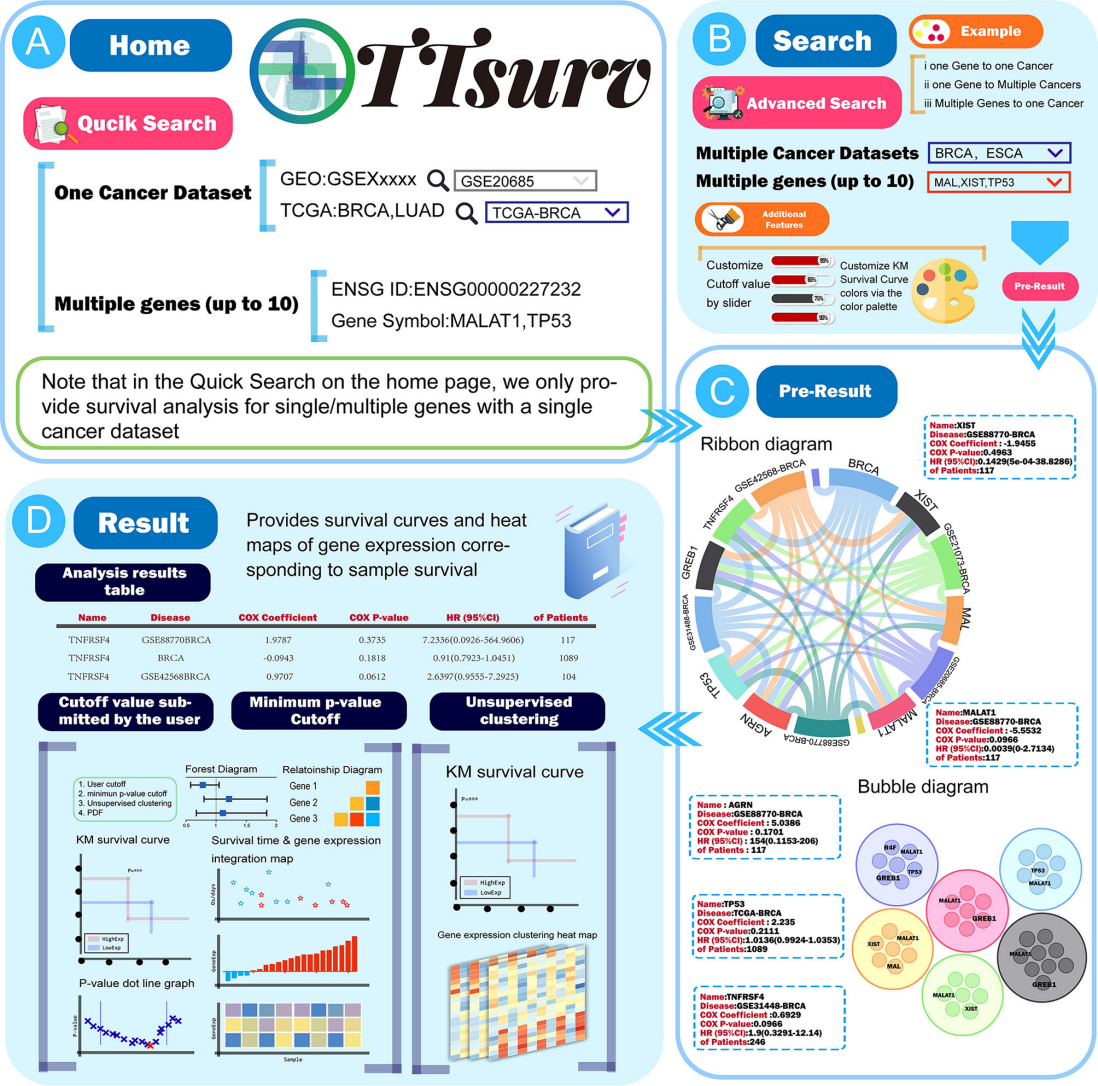
The use process and a demonstration of the results provided by TTSurv. **(A)** The data source for TTSurv and the usage of QucikSearch. **(B)** Introduction to advanced search in the search interface. **(C)** Two kinds of charts in Pre-Result, where users can get the analysis results by clicking parts of the chart. **(D)** Schematic diagram of the data table and images in the result page.

On the home page of TTSurv (**Figure 2A**), users can access the quick search page *via* the menu bar above or the quick-start button on the scroll bar of the home page (**Figure 2B**). The quick search only provides a matching mode for single or multiple genes (it should be noted that the maximum number of multi-gene queries is 10) with a single cancer dataset ID to query the relationship between the gene of interest and the target cancer dataset. When using the Quick Search function on the homepage, users need to click the Analyze button and then click “View Result” to return to the homepage to view the results. We have adopted three methods to display the pre-results in the result display screen. The user can click on the gene/dataset name in the ribbon diagram to see “Gene-all diseases/” or “Disease-all genes” (**Figure 2C**), click on the edge of the diagram to get a single gene-disease association, or click on the outer part or the edges of the graph to obtain a single gene-disease association. On the force-directed graph, the user can click on a node in a force-directed single disease and gene. On the bubble diagram, the user can click on the nodes in the bubble diagram to obtain the results of the survival analysis for the specific genes in the corresponding diseases (**Figure 2D**).

### Search Interface and Results Presentation

**Figure 3:**
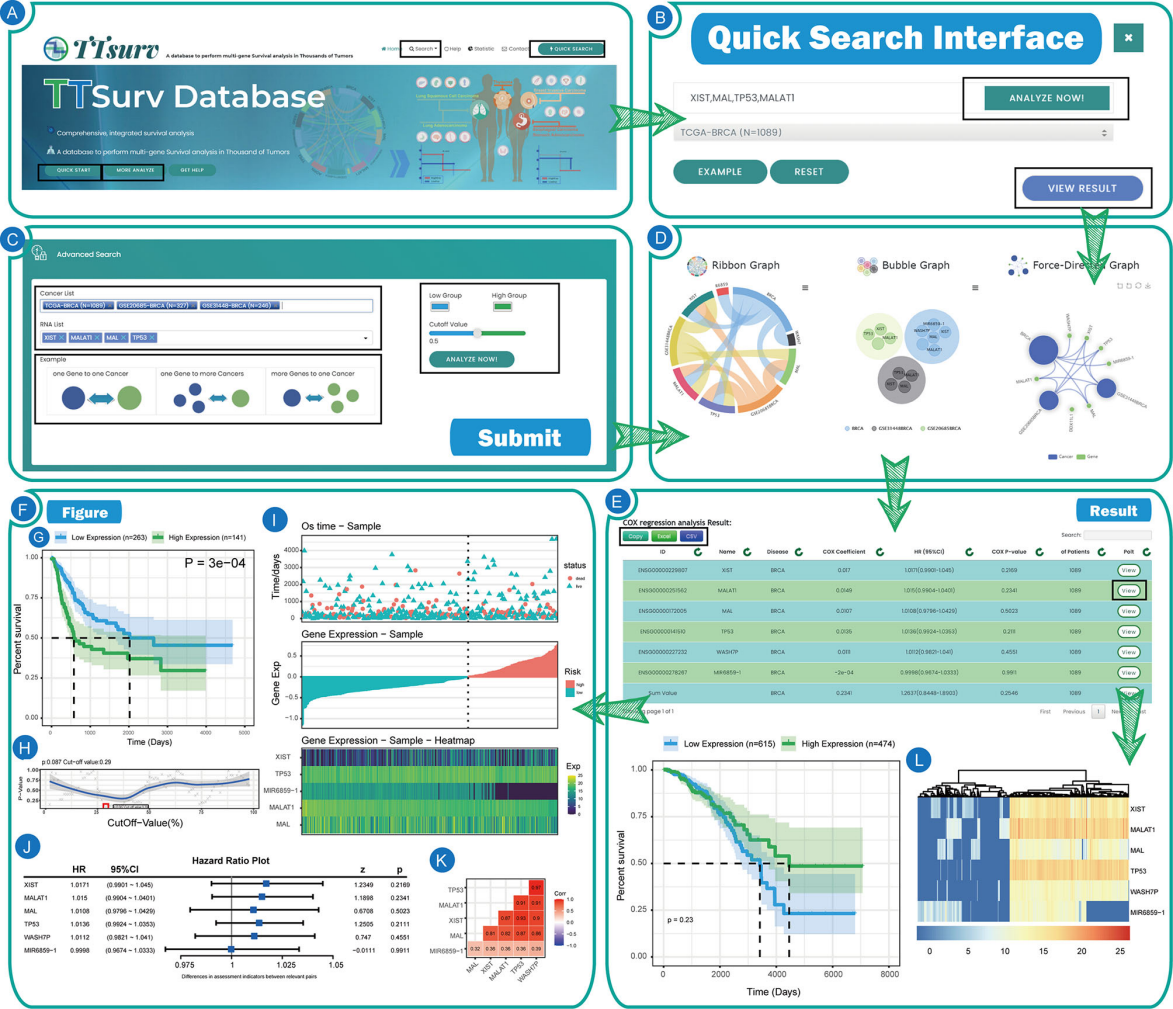
The user interfaces and results of TTSurv. **(A)** Home page of TTSurv. **(B)** Quick search interface of TTSurv. **(C)** Query interface of TTSurv. **(D)** Pre-Result interface of TTSurv. **(E)** The result table and figures of TTSurv. **(F)** The diagram display of TTSurv. **(G)** The KM survival curve map. **(H)** The P-value curve obtained after each grouping. **(I)** The Survival time & gene expression integration map. **(J)** The forest plot. **(K)** The gene correlation plot. **(L)** The heat map obtained by unsupervised clustering.

Users can access the search interface through “more analysis” in the home page rotation or by using the “Search” button in the upper menu where we additionally provide a multi-gene-multi-disease query (**Figures 3A, B**). Through this interface (**Figure 3C**), users can click the “Example” button to view the preset results for single disease-single gene/single disease-multiple gene/multiple disease-multiple genes, or query by clicking the dataset name in the Cancer List and entering the gene name in the RNA List. In this interface, we provide additional features for users to change the color of high/low expression lines in the survival plot and submit user-defined group cut values. Similarly, after clicking “Analyze” in the search interface, you will get the same result display interface as the home page and it can be used in the same way as the home page (**Figure 3D**). In the result display page, we provide the table of analysis results and three types of graphs, and users can easily access the results by using the “Copy,” “Excel” and “CSV” buttons above. In the analysis results, we provide the Gene Symbol, the Ensembl ID, the name of the disease queried, and the corresponding dataset name with the number of samples in the dataset. The results of the Cox survival analysis, including COX-P, HR values, and the COX coefficient, are also included. Users can obtain the resulting graph by clicking the “View” button on the right side of the table. In addition, users can view the results of (1) sample grouping by self-submitted cutoff values, (2) sample grouping by cutoff values calculated using the minimum p-value algorithm, and (3) sample classification by unsupervised clustering (**Figure 3E**).

### Flexible Classification

User-submitted cutoff values. Typically, users are used to grouping samples using mathematically meaningful split values (e.g., quartiles, means, and medians), and we also provide this feature to explore more possibilities (**Figure 3F**). **Figure 3G** represents the Kaplan-Meier survival analysis of the samples after grouping the sample set according to the segmentation points uploaded by the user and **Figure 3H** represents the p-value and the segmentation value obtained at each step when the samples are grouped step by step. **Figure 3I** consists of three parts. The first part is the survival time of each sample and the second part represents the gene expression of the target gene in each sample. The gene expression and risk status of the sample can be used to intuitively find whether the gene is a protective/risk factor for that type of disease. The third part shows gene expression as a heatmap, which is used to visually compare the gene expression and the survival of the patient in the first part. **Figure 3J** shows a forest plot drawn from the HR values of all incoming genes in a particular cancer dataset and **Figure 3K** shows the correlation coefficients between all genes in the same data set.

The cutoff value was obtained by the minimum p-value method. The image displayed in this result is the same as the cutoff value submitted by the user. It should be noted that the separator value of this result was calculated using the minimum p-value algorithm.

The results were obtained using unsupervised clustering for sample grouping. Unsupervised clustering can also be used to group the samples into high/low expression values based on gene expression. In this interface, we provide not only the KM curves but also a heat map of the expression profile after clustering (**Figure 3L**).

### Example Application

**Figure 4:**
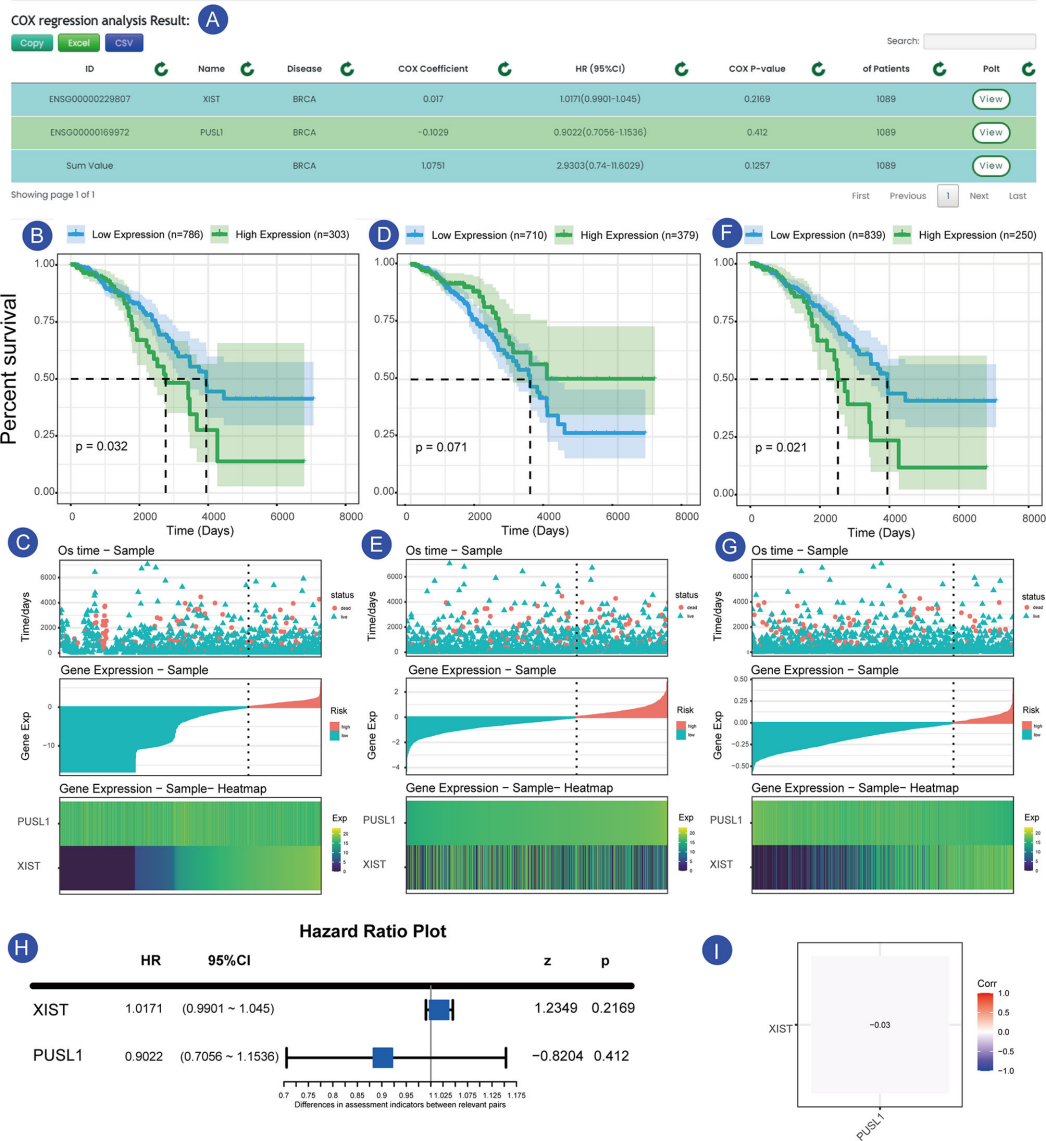
Prognosis of XIST and PUSL1 in the sample set GSE42568. **(A)** shows the results of the prognostic analysis of XIST and PUSL1. **(B, C)** show the KM survival curves and survival time-gene expression association plots for XIST, respectively. **(D, E)** show KM survival and the survival time-gene expression association plots for PUSL1, respectively. **(F, G)** KM survival curves and survival time-gene expression association plots for the SUM group (integrated data for XIST and PUSL1), respectively.Forest plot of the two genes in the breast cancer sample set. **(H)** Expression correlation plot between the XIST and PUSL1 genes, which shows that the two genes affect the survival status of patients independently of each other **(I)**.

As shown in the **Figures 4A–I**, we searched the search interface for the survival of XIST and PUSL1 in GSE42568 (breast cancer expression profile) and found that the HR value of XIST was <1, while the Cox p-value was less than 0.05. These values indicated that it was a plausible protective factor, which has been confirmed in previous experiments (17, 18). In contrast, the expression profile of the PUSL1 gene was opposite to that of the XIST gene, while a Cox-p<0.05, indicates that it is a plausible risk factor (**Figure 4A**). **Figure 4C** shows that when the samples were divided into two groups according to gene expression, the samples in the high expression group had a longer survival time and a lower mortality rate. The difference between the two groups with different expressions was more significant when the samples were divided into two parts by the minimum p-value. In **Figure 4C**, although more samples labeled as “dead” were clustered in the low XIST expression samples, the low expression group had a higher five-year survival rate (52.1%) relative to the XIST high expression samples (7.9%), which confirms the above results that XIST is a risk factor in this dataset. Currently, only a few studies have focused on the PUSL1 gene and our survival analysis shows that it may be linked to the prognosis for breast cancer, which will help researchers discover new biomarkers for cancer. Similarly, we can click on the “Sum” row results to see the overall impact of the gene set on the survival of the sample, and in this overall analysis we can see that the combined effect of XIST and PUSL1 can have a more detrimental effect on patients. This result allows us to determine which effect is greater when protective and risk factors act together in the same patient. **Figure 4G** shows the overall expression of this gene, and we can see that the samples have higher mortality and a shorter survival time when the overall gene expression trend is high, which is consistent with the results obtained from the survival time graph and the KM curve.

**Figure 5:**
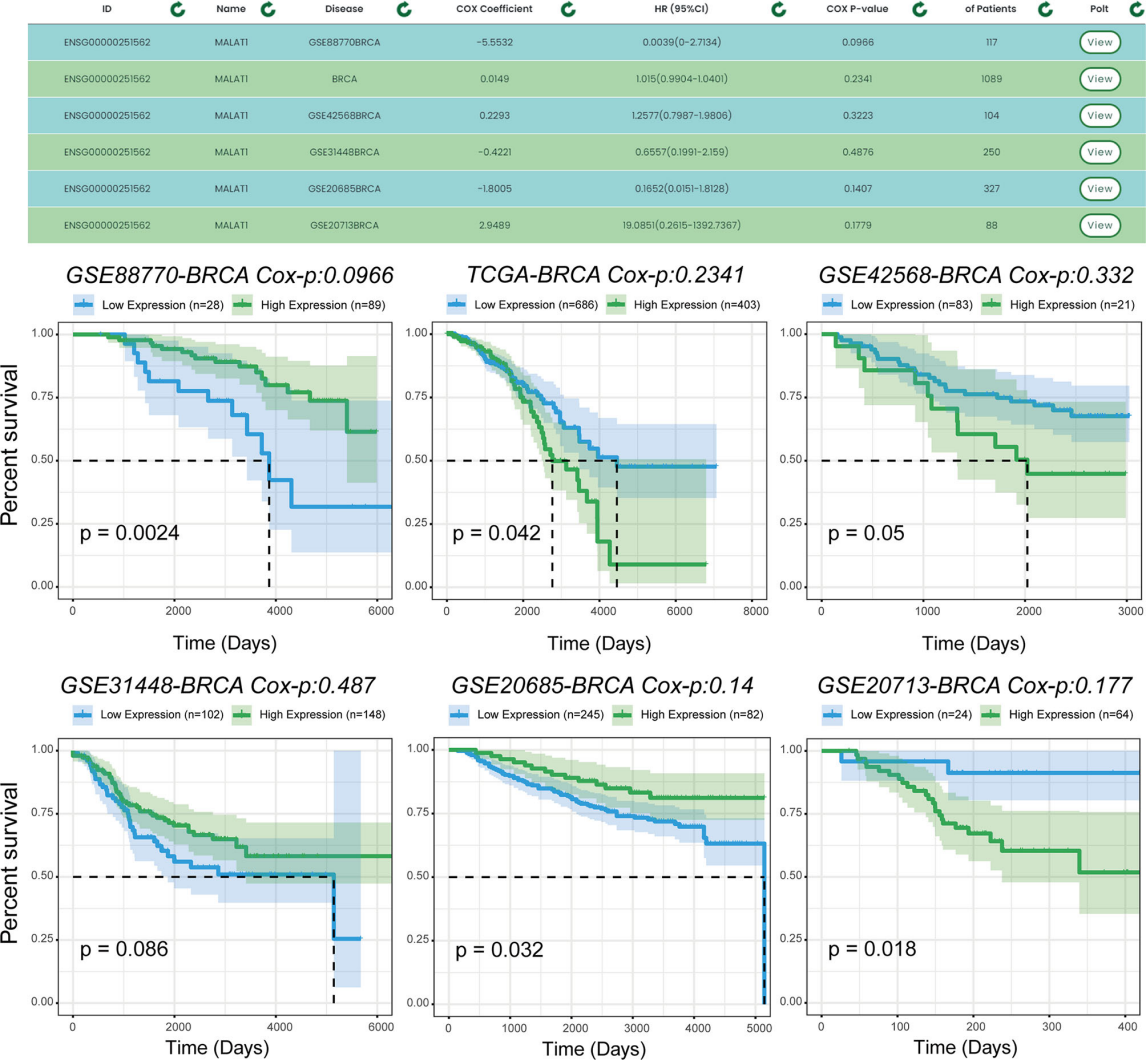
Prognostic analysis of MALAT1 in multiple data sets. MALAT1 shows different prognoses in different datasets of the same cancer, and users can use the results to make comprehensive and objective conclusions.

We used another example to illustrate the single-gene multi-disease analysis function provided by TTSurv. We found that the same gene is expressed differently in different cancers and in different datasets for the same cancer in terms of prognosis (**Figure 5**). Previous experiments have verified that MALAT1 plays a key role in breast cancer. For example, MALAT1 inhibits breast cancer metastasis (19) and MALAT1 promotes angiogenesis in breast cancer (20). However, the results obtained after the survival analysis were different. MALAT1 did not show a correlation with breast cancer prognosis in all datasets, indicating that the selection of the dataset can have an impact on the prognostic analysis results for this gene in cancer, and demonstrates the necessity of using multiple sample sets. For example, in the TCGA-BRCA and GEO-GSE42568-BRCA datasets, the risk values for MALAT1 were different, indicating that different samples have an impact on the survival analysis. By providing multiple sets of samples, we are able to provide users with a more general analysis of whether there is an association between the target gene and a particular cancer, thus providing a more comprehensive and objective analysis.

**Figure 6:**
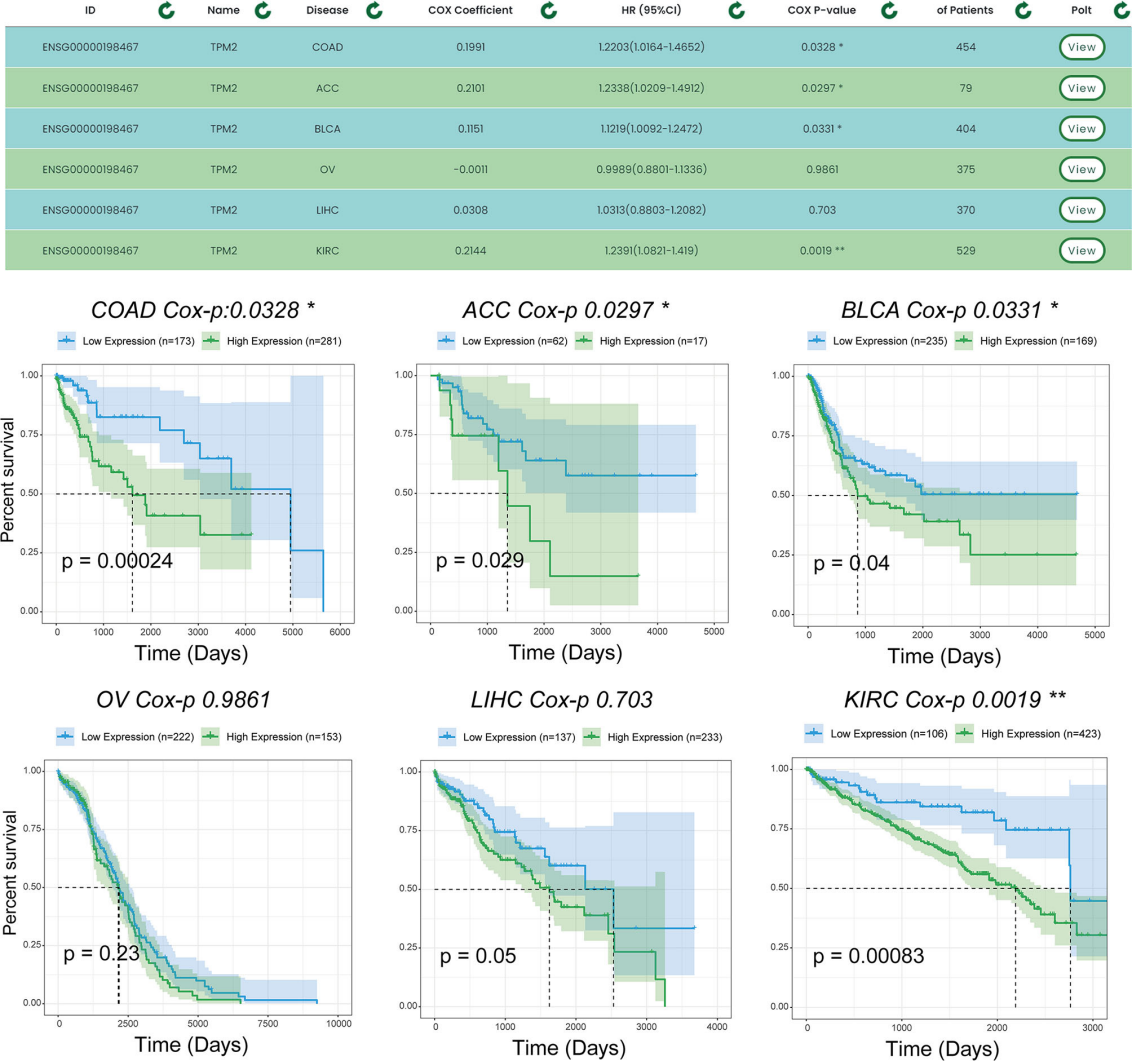
Survival analysis results for TPM2 in multiple datasets. Users can analyze the survival of a gene for pancreatic cancer using the single gene-multiple disease function. “*” indicates p-value <0.05, “**” indicates p-value <0.01.

To demonstrate the single-gene multi-disease survival analysis function, we show the effect of the TPM2 gene on patient survival status in six cancer datasets (which were ‘TCGA-COAD’, ‘TCGA-ACC’, ‘TCGA-BLCA’, ‘TCGA-OV’, ‘TCGA-LIHC’, and ‘TCGA-KIRC’). **Figure 6**, shows the results of the survival analysis with survival curves for TPM2 in six cancer datasets. Similarly, TPM2 has been demonstrated in previous experiments to be a diagnostic marker for colorectal cancer and breast cancer. For example, hypoxia-induced TPM2 methylation is associated with chemoresistance and poor prognoses for breast cancer (21), and is also associated between epigenetic silencing of TPM2 and colorectal cancer (22). TPM2 has also been found to have potential as a diagnostic marker for patients with adrenocortical carcinoma as well as bladder urothelial carcinoma, and similar findings will provide guidance to researchers.

## Discussion

There are already several online tools capable of performing online survival data analyses, including Kaplan-Meier Plotter (23), GENT (24) and ITTACA (25). TTSurv improves the functionality of previously published databases, which often lack more flexible grouping algorithms and a comprehensive analysis and presentation of results. For example, although the Kaplan-Meier Plotter database can automatically select the best cutoff value, it cannot provide results other than survival curves and cutoff plots. With the increasing abundance of clinical data and high-throughput data, biomarkers associated with cancer patient survival will be confirmed by more comprehensive survival analysis studies. TTSurv aims to discover biomarkers closely associated with patient survival status and provide support for the analysis results through the collection and integration of public data and analysis. The biomarkers we provide can be used to reveal individual pathologies and drive the development of precision medicine research. Most online analysis tools tend to give one-sided results (including inadequate legends and grouping) and are dependent on data volume.

In the future, we plan to collect more data samples and provide improved functionality, and we will continue to improve our database in the following areas: (i) collecting more newly released datasets for thoracic tumors; (ii) adding more visualizations: e.g., forest plots based on their HR values in single-gene multi-datasets; and (iii) improving the annotation of probes that cannot be annotated in the current dataset. We believe that TTSurv will be a useful resource for researchers at many stages from target discovery to target validation through continuous updates. We believe that through continuous updates, TTSurv will become an important online survival analysis tool and provide researchers with powerful aid in a variety of ways.

## Conclusions

TTSurv is unique because it can group samples in multiple ways to find more possible associations between target genes and cancer. We provide an integration algorithm that analyzes the set of user-submitted genes as a whole for a more comprehensive analysis and more valuable conclusions. At the same time, the multiple outcome data we provide offer strong support for the augmentation of gene-cancer associations. We hope that TTSurv will be a useful resource for cancer researchers at multiple stages, from target discovery to target validation.

## Data Availability Statement

Publicly available datasets were analyzed in this study. This data can be found here: http://www.bio-server.cn/TTSurv.

## Author Contributions

YQ, MX, YZ and QG conceived and designed the experiments. YH, QL, and PW analyzed data. YQ and MX collected the data. YH and PW validated the method and data. YQ and QG wrote this manuscript. All authors contributed to the article and approved the submitted version.

## Funding

This work was supported by the National Natural Science Foundation of China [81902646 and 32070622], Heilongjiang Provincial Natural Science Foundation [LH2020H046]; Postdoctoral Science Foundation of China [2020M670922]; Postdoctoral Foundation of Heilongjiang Province [LBH-Z19077 and LBH-Q20047].

## Conflict of Interest

The authors declare that the research was conducted in the absence of any commercial or financial relationships that could be construed as a potential conflict of interest.
