# Supplementary material for: TTSurv: Exploring the Multi-Gene Prognosis in Thousands of Tumors
Source: Front Oncol. 2021 May 25;11:691310. doi: 10.3389/fonc.2021.691310 (PMC8186665; doi:10.3389/fonc.2021.691310)
Supplement: Supplementary file 1 [file Table_1.docx]

**Table S1：Samples and clinical information from the TCGA database for 33 cancer types**

| Cancer Type | Description | Samples |
| --- | --- | --- |
| ACC | Adrenocortical carcinoma | 92 |
| BLCA | Bladder Urothelial Carcinoma | 404 |
| BRCA | Breast invasive carcinoma | 1089 |
| CESC | Cervical squamous cell carcinoma and endocervical adenocarcinoma | 304 |
| COAD | Colon adenocarcinoma | 454 |
| ESCA | Esophageal carcinoma | 163 |
| GBM | Glioblastoma multiforme | 159 |
| HNSC | Head and Neck squamous cell carcinoma | 500 |
| KICH | Kidney Chromophobe | 65 |
| KIRC | Kidney renal clear cell carcinoma | 529 |
| KIRP | Kidney renal papillary cell carcinoma | 289 |
| LAML | Acute Myeloid Leukemia | 151 |
| LGG | Brain Lower Grade Glioma | 506 |
| LIHC | Liver hepatocellular carcinoma | 370 |
| LUAD | Lung adenocarcinoma | 515 |
| LUSC | Lung squamous cell carcinoma | 497 |
| MESO | Mesothelioma | 85 |
| OV | Ovarian serous cystadenocarcinoma | 375 |
| PAAD | Pancreatic adenocarcinoma | 177 |
| PCPG | Pheochromocytoma and Paraganglioma | 179 |
| PRAD | Prostate adenocarcinoma | 475 |
| READ | Rectum adenocarcinoma | 166 |
| SARC | Sarcoma | 257 |
| SKCM | Skin Cutaneous Melanoma | 456 |
| STAD | Stomach adenocarcinoma | 380 |
| TGCT | Testicular Germ Cell Tumors | 133 |
| THCA | Thyroid carcinoma | 502 |
| THYM | Thymoma | 119 |
| UCEC | Uterine Corpus Endometrial Carcinoma | 542 |
| UCS | Uterine Carcinosarcoma | 56 |
| UVM | Uveal Melanoma | 80 |

* We filtered low sample size data

**Table S2：Samples and clinical information from cancer datasets in the GEO database**

| Cancer Type | Description | Samples |
| --- | --- | --- |
| BRCA | Breast Invasive Carcinoma | GSE2603 (N=82) |
| BRCA | Breast Invasive Carcinoma | GSE5327 (N=58) |
| BRCA | Breast Invasive Carcinoma | GSE6130 (N=107) |
| BRCA | Breast Invasive Carcinoma | GSE10886 (N=101) |
| BRCA | Breast Invasive Carcinoma | GSE10893 (N=237) |
| BRCA | Breast Invasive Carcinoma | GSE18229 (N=253) |
| BRCA | Breast Invasive Carcinoma | GSE20685 (N=327) |
| BRCA | Breast Invasive Carcinoma | GSE20713 (N=88) |
| BRCA | Breast Invasive Carcinoma | GSE21653 (N=252) |
| BRCA | Breast Invasive Carcinoma | GSE31448 (N=246) |
| BRCA | Breast Invasive Carcinoma | GSE37181 (N=123) |
| BRCA | Breast Invasive Carcinoma | GSE42568 (N=104) |
| BRCA | Breast Invasive Carcinoma | GSE48390 (N=81) |
| BRCA | Breast Invasive Carcinoma | GSE58812 (N=107) |
| BRCA | Breast Invasive Carcinoma | GSE88770 (N=117) |
| ESCA | Esophageal Carcinoma | GSE53622 (N=60) |
| ESCA | Esophageal Carcinoma | GSE53624 (N=119) |
| LUAD | Lung Adenocarcinoma | GSE3141 (N=111) |
| LUAD | Lung Adenocarcinoma | GSE11969 (N=149) |
| LUAD | Lung Adenocarcinoma | GSE31210 (N=226) |
| LUAD | Lung Adenocarcinoma | GSE26939 (N=115) |
| LUAD | Lung Adenocarcinoma | GSE30219 (N=293) |
| LUSC | Lung Squamous Cell Carcinoma | GSE8894 (N=138) |
| LUSC | Lung Squamous Cell Carcinoma | GSE19188 (N=82) |
| LUSC | Lung Squamous Cell Carcinoma | GSE37745 (N=196) |
| LUSC | Lung Squamous Cell Carcinoma | GSE50081 (N=181) |
| LUSC | Lung Squamous Cell Carcinoma | GSE42127 (N=176) |
| STAD | Stomach Adenocarcinoma | GSE15459 (N=192) |
| STAD | Stomach Adenocarcinoma | GSE62254 (N=300) |
| BLCA | Bladder Urothelial Carcinoma | GSE13507 (N=165) |
| COAD | Colon Adenocarcinoma | GSE38882 (N=122) |
| OV | Ovarian Serous Cystadenocarcinoma | GSE14764 (N=80) |
| OV | Ovarian Serous Cystadenocarcinoma | GSE17260 (N=110) |
| OV | Ovarian Serous Cystadenocarcinoma | GSE26712 (N=56) |
| OV | Ovarian Serous Cystadenocarcinoma | GSE30161 (N=58) |
| OV | Ovarian Serous Cystadenocarcinoma | GSE31245 (N=57) |
| OV | Ovarian Serous Cystadenocarcinoma | GSE32602 (N=260) |
| OV | Ovarian Serous Cystadenocarcinoma | GSE49997 (N=194) |
| OV | Ovarian Serous Cystadenocarcinoma | GSE63885 (N=70) |
| PRAD | Prostate Adenocarcinoma | GSE16560 (N=281) |
